# Zero–Waste Recycling of Fiber/Epoxy from Scrap Wind Turbine Blades for Effective Resource Utilization

**DOI:** 10.3390/polym14245408

**Published:** 2022-12-10

**Authors:** Chunbao Du, Ge Jin, Lihui Zhang, Bo Tong, Bingjia Wang, Gang Zhang, Yuan Cheng

**Affiliations:** 1 College of Chemistry and Chemical Engineering, Xi’an Shiyou University, Xi’an 710065, China; 2 Shaanxi Engineering and Technology Research Center of Green Low—Carbon Energy Materials and Processes, Xi’an 710065, China; 3 Xi’an Thermal Power Research Institute Co., Ltd., Xi’an 710054, China; 4 Institute of High Performance Computing, A*STAR, Singapore 138632, Singapore; 5 Monash Suzhou Research Institute, Monash University, Suzhou Industrial Park, Suzhou 215000, China; 6 Department of Materials Science and Engineering, Monash University, Clayton, VIC 3800, Australia

**Keywords:** scrap wind turbine blade, epoxy resin/glass fiber, pyrolysis, resource utilization, flexible composite

## Abstract

The number of scrap wind turbines is expanding globally as the wind power industry develops rapidly. Zero–waste recycling of scrap wind turbine blades (WTB) is the key for wind power firms to achieve green and sustainable development on the premise of satisfying environmental protection criteria. In this work, the pyrolysis of fiber/epoxy composites obtained from scrap WTB in oxidizing inert atmospheres was investigated. Various characterization methods were employed to characterize the microstructure and chemical characteristics of the heat–treated fiber/epoxy and to reveal the pyrolysis mechanism. In addition, the heat–treated fibers/epoxy were used as reinforcing agents to investigate their impact on the elastic deformation of butadiene styrene rubber–based flexible composites, and the reinforcing mechanism was revealed. The results revealed that the constituents of fiber/epoxy composites were mostly fiberglass (SiO_2_, CaCO_3_) and cured epoxy resin, with covalent bonding being the interaction between the fiberglass and epoxy resin. The total weight of the epoxy resin in the fiber/epoxy composites was 22%, and the 11% weight loss was achieved at around 350 °C, regardless of the presence of oxygen; however, the features of heat–treated fibers/epoxy were associated with the pyrolysis atmosphere at a higher temperature. The pyrolysis products in inert atmospheres, with water contact angles of 58.8°, can considerably improve the tensile properties of flexible composites at the elastic stage. Furthermore, the flexible composite granules were prepared to plug large channels in sand–filled pipes, and the plugging rate had the potential to reach 81.1% with an injection volume of 5.0 PV. The plugging performance was essentially unaffected by water salinity, owing to the high stability of flexible composite granules in mineralized water. The findings of this study present a realistic route to the industrial application of fiber/epoxy, as well as a novel approach for encouraging the efficient use of scrap wind turbines on a large scale.

## 1. Introduction

Wind power generation is a type of green power generation that helps to promote the global clean energy transition, reduce carbon dioxide emissions, and solve the climate change challenge [1,2]. Due to the 20 year lifespan of wind turbines, which is by design, decommissioning wind turbines has been a major challenge for the global wind power sector as a result of the exponential growth of wind power usage [3,4]. Fiber/epoxy composites are produced by curing epoxy resin on fibers, and they have the most value both technically and in terms of market innovation. As the scale and demand for fiber/epoxy composites rise in the wind power industry, the scrap peak for fiber/epoxy composite products approaches, thus resulting in a significant increase in scrap volume [5,6]. Due to the stable three–dimensional network structure, these waste fiber/epoxy composites are difficult to recycle and they end up as “stubborn” pollutants that seriously pollute the environment [7,8,9]. The epoxy resin and fibers in WTB contribute up to 9% of the weight of the WTB, and they are predicted to reduce carbon emissions by 8.1%; therefore, the fiber/epoxy composites have become the key to the wind power industry’s recovery and the green and low–carbon image of the industry.

At present, the chemical recovery of waste fiber/epoxy composites is mostly focused on the recovery of reinforcing materials (carbon fiber, glass fiber, etc.) from composites due to the high cost of recycling resin [10,11,12]. The cause of these phenomena is that most degradation processes employ extremely corrosive acids (e.g., nitric acid, sulfuric acid, etc.) or alkalis as catalyst systems which are used in high pressure environments [13,14]. Harsh degradation conditions and complicated processes cause polymers to decompose into complex mixes of gases, liquids, and solids, thus making reusing these materials challenging. Furthermore, the recovered reinforcing material would be damaged, thus leading to a poor performance [15].

Pyrolysis has long been seen as a potential technology for the clean and effective exploitation of high–quality organic solid waste, capable of converting organic solid waste into carbon material or liquid for chemical raw materials [16,17]. A number of investigations on the pyrolysis of fiber/epoxy composites has been conducted by researchers. Regnier et al., for example, investigated the pyrolysis kinetics of carbon fiber/epoxy composites and estimated their apparent activation energies at various stages of decomposition [18]. Pickering et al. employed a fluidized bed to heat the waste of a thermosetting resin composite material at 450 °C and a 1.3 m/s air flow rate, and they also used a rotary filtering separator to obtain glass fibers and mineral fillers of various lengths [19]. Naqvi et al. reviewed the research progress of the pyrolysis waste recycling of fiber/epoxy composites in order to realize circular economy [20]. At present, more research is needed to evaluate the stepwise pyrolysis process for various types of fiber/epoxy composites. Furthermore, a thorough examination of the economic and energy analyses of pyrolysis processes is currently lacking in the literature; this information would demonstrate the environmental impact of pyrolysis products as well as the economic benefits of avoiding end–of–life landfill. Guo et al. recently performed a two–step pyrolysis and oxidation process study on carbon fiber/epoxy composites and found that the tensile modulus and conductivity of carbon fibers produced through pyrolysis at 450 °C in an aerobic environment were greatly improved, owing to the high temperature pyrolysis products containing 3–methyltetrahydrophthalic anhydride, bisphenol A, and other oxygen–containing aromatic compounds [21]. The carbon transformed by epoxy resin can be deposited on carbon fibers by controlling the pyrolysis conditions, which has significant advantages for further improving the performance of carbon fibers; however, the deposition of pyrolysis products on epoxy resin on glass fibers has rarely been reported. Furthermore, it is impossible to separate and recycle epoxy resin and glass fibers that are on glass fiber/epoxy composites using basic physical or chemical procedures. Epoxy resin has a higher recycling value than glass fiber due to its higher carbon content; however, because glass fiber has a temperature resistance of more than 800 °C, and epoxy resin has a temperature resistance of less than 600 °C, it is not possible to obtain an epoxy resin resource without any glass fiber. Due to the aforementioned conflicts, there are limited reports on how to use the waste glass fiber reinforced epoxy resin matrix composites that are produced during pyrolysis.

Due to the substantial disorder within the pulverized structure after mechanical pulverization, scrap WTB may not be acceptable for high–end application in industry. As the global yearly scrap volume of WTB is so great, it is better suited to more practical industrial uses. Scrap WTB powder is most likely to be used as cement filler in the building sector, but this application has several limitations [22,23]. The inclusion of fibers/epoxy, for example, has no considerable performance gain, and the function of epoxy containing carbon cannot be completely used. By 2050, the globe will have 43 million tons of scrap WTB, and the fiber/epoxy composites in scrap WTB are challenging to recycle and utilize on a large scale [24].

Given the global need for energy and governments’ concerns about energy security, the development of oil and gas resources is a field requiring long–term study [25,26]. The majority of oilfields in the world are maintained by water injection, which results in significant reservoir heterogeneity and seepage channels [27]. Injected water flows via formation cracks and large, deep holes result in the inefficient or invalid circulation of injected water, which has a significant impact on the water flooding effect [28]. As a result, deep profile control technology must be used to optimize the water absorption profile, raise the water flooding sweep coefficient, and improve oil recovery [29,30]. The existing profile control system cannot simultaneously meet the characteristics of good deformation ability and high temperature and salt resistance in an adverse formation environment; therefore, it cannot effectively improve the heterogeneity of deep formation, thus resulting in low oil recovery [31]. Although scrap WTB powder has the potential to increase the temperature resistance of profile control agents, no studies have been conducted in this area thus far.

In this study, the waste fiber/epoxy composites were ground and then thermally treated to investigate the pyrolysis process of waste fibers/epoxy in oxidizing inert atmospheres. Thereafter, the pyrolysis products were used as reinforcement agents to improve the elastic deformation of flexible materials. In addition, the application potential of flexible composite granules as plugging agents of large channels was studied via granulation with a twin screw extruder in order to expand the possibility of their large–scale application. This study proposes a novel method for recycling scrap WTB in its entirety instead of recycling the resin or glass fiber separately.

## 2. Materials and Methods

### 2.1. Experimental Materials

Scrap bulk WTB (Figure S1a) were obtained from Xi’an Thermal Power Research Institute Co., LTD. (Xi’an, China). Styrene–butadiene rubber (SBR, M_n_: 100000) was purchased from the China Petrochemical Corporation. Lightweight liquid paraffin and NaCl (AR) were purchased from Shanghai Aladdin Biochemical Technology Co., LTD. (Shanghai, China).

### 2.2. Pyrolysis of Fibesr/Epoxy

The scrap WTB were first physically cut and ground using a grinding machine. Then, the obtained powder was sifted with a 20 mesh sieve and labeled as fiber/epoxy. The sifted fibers/epoxy (Figure S1b) were put in a Muffle furnace (DC–B5, DOTRUST) and heated to 450 °C at a heating rate of 10 °C min^−1^. After pyrolysis for 1 h, the obtained sample was labeled as fiber/C1 (Figure S1c). Similarly, the fibers/epoxy were placed in a tubular furnace (CY–O1200–50IL, CY Scientific Instrument Co., Ltd., Zhengzhou, China) and then oxygenated with N_2_ for 15 min. Subsequently, the tubular furnace was heated to 450 °C at 10 °C min^−1^, and then held for 1 h. The obtained sample in the N_2_ atmosphere was labeled as fiber/C2 (Figure S1d).

### 2.3. Preparation of the Flexible Composite

The SBR, liquid paraffin, and reinforcing agent, with a mass fraction of 1: 10: 8, were added into the inner lining of a miniature high–pressure reaction kettle (LB250, Shanghai Laibei Scientific Instrument Co., Ltd., Shanghai, China). The mixture was placed in a high temperature, high pressure reaction kettle after being uniformly agitated with a glass rod. The flexible composite was produced after stirring at 180 °C for 2 h. To produce the molded flexible composite for mechanic performance testing, the flexible composite was cut into pieces, put into a dumbbell mold (50 × 8.5mm), and extruded at 180 °C on a high temperature hot table. After compaction and chilling, dumbbell–shaped samples were produced. The flexible composites that were made with fibers/epoxy, fiber/C1 and fiber/C2, were reinforcing agents that were labeled fiber/epoxy@FC, fiber/C1@FC, and fiber/C2@FC, respectively.

### 2.4. Preparation of Flexible Composite Granules

The flexible composite granules have the same composition as the flexible composite; however, their form and size were different. To prepare flexible composite granules using fiber/C2 as reinforcing agent, the SBR, liquid paraffin, and fiber/C2 with a mass fraction of 1:10:8 were added into the barrel containing the twin screw extruder (Dongguan Chengtai Automation Technology Co., Ltd., Dongguan, China). The reaction temperature of the twin screw, the grinding head temperature, and the underwater cutting temperature were 180, 140, and 60 °C, respectively. After dehumidifying using a vibrating screen and fan, the flexible composite granules were obtained. The average size of the flexible composite granules was controlled by the size of the grinding head.

### 2.5. Characterization

An X–ray diffractometer (XRD, D8 Advance, Bruker, Billerica, MA, USA) was used to characterize the components of the fibers/epoxy before and after pyrolysis. To analyze the elements and chemical states of the fibers/epoxy, X–ray photoelectron spectroscopy (XPS, Shimadzu Kratos, Manchester, UK) was utilized. A field emission scanning electron microscope (SEM, Talos F200X, FEI, Lausanne, Switzerland) was used to analyze the microstructure of the fibers/epoxy before and after pyrolysis. A synchronous thermal analyzer (TGA/DSC1, METTLER TOLEDO, Columbus, OH, USA) was used to evaluate the thermal characteristics of the fibers/epoxy under various heat treatment settings. The tensile characteristics of the flexible composites at various temperatures were characterized using a universal testing machine (CMT6104, MTS, Eden Prairie, MN, USA). The digital rubber hardness tester was used to assess the hardness of the flexible composite granules. The aging properties of the flexible composite granules were studied after soaking in a NaCl solution (200 g L^−1^) for 3 months.

### 2.6. Plugging Experiment

**Scheme 1:**
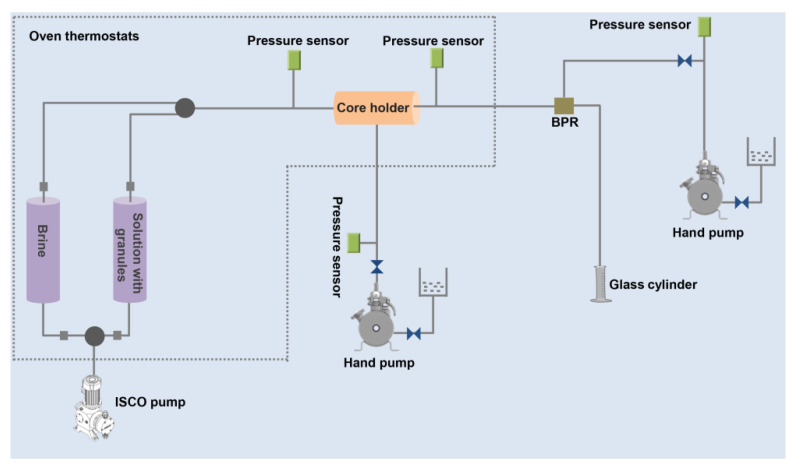
Schematic of the sandpack flooding experimental setup.

A sand–filled pipe simulation system (Scheme 1) was used to investigate the plugging performance of flexible composite granules for large channels. The pipe with a length of 30 cm was filled with dried quartz sand (50~120 mesh size). The sand–filled pipe was saturated with deionized (DI) water, and the injection rate was set at 1 mL min^−1^. The sand–filled pipe’s initial permeability was determined to be *k*_1_ (mD). Flexible composite granules (0.5 wt%) were injected into the simulated mineralized water with a NaCl concentration of 50~200 g L^−1^; the injected rate was 1 mL min^−1^. The flow and pressure changes were measured during the injection process. After the pressure remained constant, the two ends of the sand–filled pipe were sealed and aged for 3 days in a 120 °C oven. Then, DI water was injected, and the breakthrough pressure, flow rate, and pressure change were recorded, as was the permeability *k*_2_ (mD) of the sand–filled pipe.

The plugging rate (*D*, %) was determined in accordance with the following equation (Equation (1)) [32]:


(1) $$D=\frac{k_{1}-k_{2}}{k_{1}}\times 100\%$$

## 3. Results and Discussion

### 3.1. Structural and Compositional Analysis of the Fibers/Epoxy

**Figure 1:**
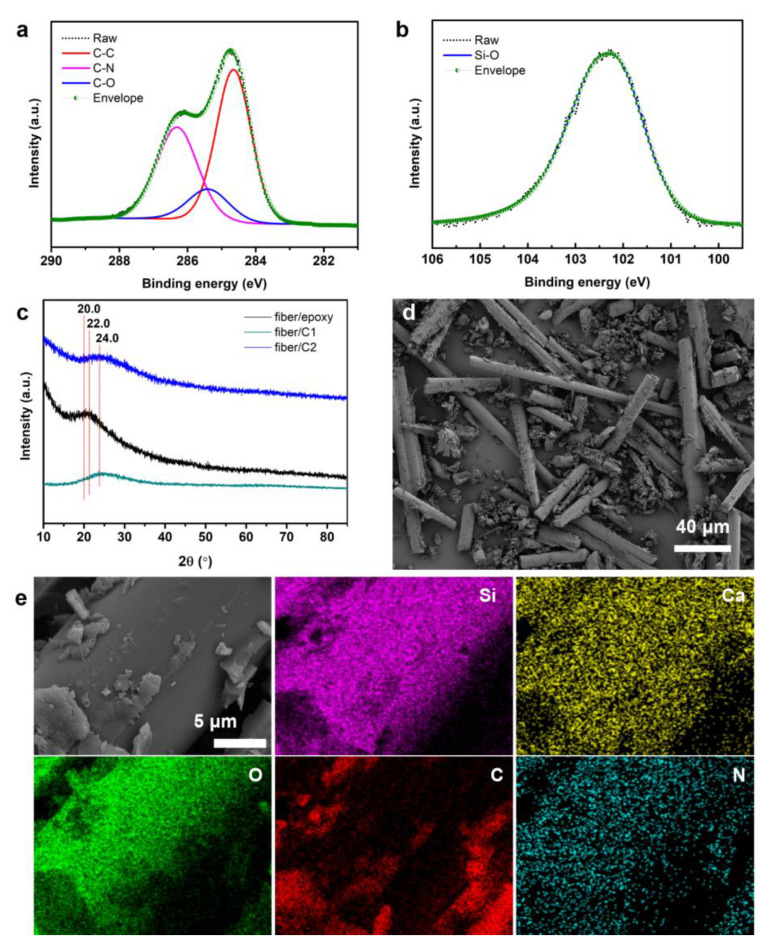
XPS survey spectra of the fibers/epoxy: C 1s (**a**), Si 2p (**b**); XRD spectra of the fibers/epoxy (**c**); SEM image of the fibers/epoxy (**d**); SEM mapping of the fibers/epoxy (**e**).

**Figure 2:**
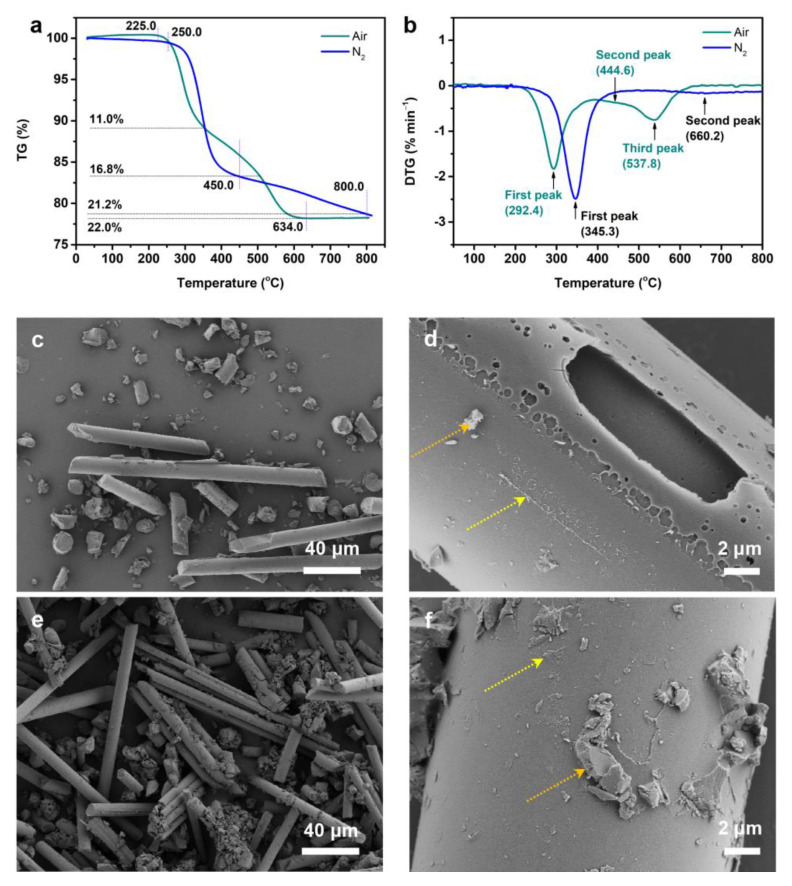
TG (**a**) and DTG (**b**) curves of fibers/epoxy under different atmospheres; SEM images of fibers/epoxy under different atmospheres: air (**c**,**d**), and N_2_ (**e**,**f**).

The main elements of the fibers/epoxy in the XPS broad spectrum were C, O, N, Si, and Ca (Figure S2). The defining elements of the epoxy resin were C, O, and N, thus suggesting that the epoxy resin was cross–linked by an amine crosslinking agent. Si, Ca, and O were the glass fiber’s distinguishing elements, thus showing that it was an inorganic nonmetallic substance with Si and Ca as its primary components. The three peaks of C 1s, with binding energies of 284.6, 285.7 and 286.5eV, respectively, were C–C, C–N, and C–O in epoxy resin (Figure 1a). In addition, there were four peaks in O 1s, with binding energies of 531.2, 532.5, 533.3, and 533.7eV, which were ascribed to Ca–O, C–O, C=O, and Si–O, respectively (Figure S4). Furthermore, N, Si, and Ca all exhibited a single chemical state, with binding energies of 399.1, 102.2, and 347.9 eV for C–N, SiO_2_, and CaCO_3_, respectively (Figure 2, Figures S5 and S6). As an amorphous polymer, epoxy resin has a distinctive diffraction peak of about 20° in the XRD pattern, whereas amorphous SiO_2_ has a characteristic diffraction peak of around 22° [33,34]. The fibers/epoxy showed a large peak at 21°, which was a superposition of the epoxy resin, and amorphous SiO_2_ diffraction peaks were also evident (Figure 1c). The distinctive peak of CaCO_3_ was not visible in the XRD pattern due to its low content in glass fiber. According to the XPS and XRD data, the primary components of the fibers/epoxy were epoxy resin with a N–containing crosslinking agent, SiO_2_, and a trace amount of CaCO_3_.

After crushing, the fibers/epoxy exhibited a characteristic fiber and detritus structure, with a fiber diameter of about 17 μm and irregular detritus (the arrows’ place) granules on the surface (Figure 1d). The varied lengths of the fibers and debris were caused by mechanical physical crushing, which occurred because the fibers/epoxy were obtained during the curing reaction between the epoxy resin and glass fibers. SEM imaging on the fibers/epoxy was performed to better identify the dispersion of the epoxy resin and glass fiber. The fibers/epoxy mainly contained C, N, O, Si and Ca, which was consistent with the XPS analysis. The length distribution of the fibers in the fiber/epoxy composite was calculated using the following equation (Equation (2)). [35]:

(2) $$\text{D=}\overset{\text{k}}{\underset{\text{i=1}}{\sum }}{\text{n}}_{\text{i}}{\text{D}}_{\text{i}}\text{/}\overset{\text{k}}{\underset{\text{i=1}}{\sum }}{\text{n}}_{\text{i}}$$

where, D is the number–average length, k is the total number of measured fibers, n_i_ is the number of determined fibers (n_i_ > 100), and D_i_ is the length of the determined fibers; the Nano Measurer 1.2.5 software determined the values of D_i_ and n_i_. It was found that the fibers which had an average length of less than 96 μm accounted for 85.1% of the total fiber content (Figure S3).

The surface elements of the fibers were mostly O, Si, and Ca, whereas the debris on the glass fiber surface was mostly composed of C and O (Figure 1e), thus indicating that the debris in the fiber/epoxy that was obtained by physical pulverizing was mostly epoxy resin. The O signal in the epoxy resin was visibly weaker than that in the glass fibers, thus suggesting that the O content of the glass fibers was significantly greater than that of the epoxy resin. The physical crushing, however, cannot completely peel the epoxy resin from the glass fiber surface, thus indicating that prior to physical crushing, the epoxy resin and glass fiber were not simply physically bound. The surface modification of glass fiber was helpful for its reaction with the epoxy precursors; this does not occur during the curing process, but with the epoxy precursor during the curing process [36].

### 3.2. TG Experiments of Fiber/Epoxy

The TG and DTG curves of fiber/epoxy degradation in air and in N_2_ atmospheres were shown in Figure 2a–b, and corresponding pyrolysis data were included in Table 1. In the air atmosphere, the fibers/epoxy exhibited a maximum weight loss of 22.0% at 800 °C; therefore, the mass ratios of epoxy resin and the glass fibers were 22.0 and 78.0%, respectively. T_0.05_, T_0.11_, and T_0.22_ denoted thermal mass losses of 5%, 11%, and 22%, respectively, whereas T_max_ represented the temperature at which the greatest decomposition rate was derived from the DTG curve. The pyrolysis of the fibers/epoxy in the N_2_ atmosphere resulted in a single weight loss, which occurred mostly at 350 °C. The TG curve, however, altered substantially when exposed to air in an oxygenated environment. In the air atmosphere, the initial decomposition of the epoxy resin in the fiber/epoxy composite happened earlier. Interestingly, an 11% mass loss of fibers/epoxy was attained at roughly 354~357 °C, without the presence of oxygen. Ma et al. attributed the first degradation step to the release of ester groups because the monomer and curing agent of the resin were methylhexahydrophthalic anhydride and N,N–Dimethylbenzylamine, respectively [16]; however, due to the uncertain components of resin in this work, the components of degradation could not be determined. The mass loss in the second stage was typically attributed to the early epoxy resin decomposition and oxidation of the resin residue [37,38]. The second and third peak positions were connected to oxygen. In the presence of oxygen, a third peak appeared at 537.8 °C, which thus corresponds with the oxidative decomposition of the residue. There are three situations involving the oxidative pyrolysis of organic polymers [39]. The first case involves a continuous process of pure pyrolysis followed by burning the resulting residue. The second case involves the in situ heterogeneous oxidation of organic solids and the final formation of combustion products. The third case involves an intermediate reaction pathway between the first case and the second case; this creates a synergistic effect of pyrolysis and heterogeneous oxidation. In the third case, moderate oxidation improved the pyrolysis process, with partial oxidation delaying further degradation of the sample. The oxidative pyrolysis of epoxy resin corresponded with the third case, thus suggesting that it cannot be described by a distinct pyrolysis or combustion model. The fibers/epoxy disintegrated completely at 634 °C, with a weight loss of 22% in the oxidizing atmosphere; conversely, in an inert atmosphere, the weight loss was 18.9% and the residue retention was 0.8%.

### 3.3. Characterization of the Fibers/Epoxy under Different Conditions

**Figure 3:**
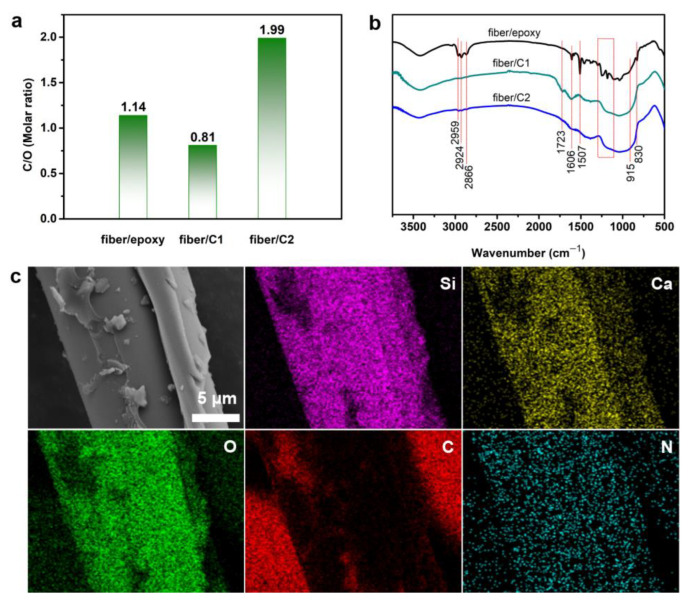
Molar ratio of C/O of the fiber/epoxy composite, fiber/C1, and fiber/C2 (**a**); FTIR spectra of the fiber/epoxy composite, fiber/C1, and fiber/C2 (**b**); SEM imaging of fiber/C2 (**c**).

Due to the thermal decomposition of the epoxy resin, the surfaces of the prepared fiber/C1 and fiber/C2 were smoother than the fiber/epoxy composite (Figure 2c–f). There was still a substantial amount of debris and exfoliated material on the fibers’ surfaces, thus implying that the epoxy resin did not fully degrade after these pyrolysis procedures; this was consistent with the results of the TG investigation. A considerable number of tiny granules appeared on the surface of the debris in the presence of O_2_ (Figure 2d), thus showing that new compounds were generated in the presence of O_2_. The free radicals formed during epoxy resin decomposition interacted with O_2_ to form stable peroxide free radicals which needed higher temperatures to decompose [37]. This was identical to the third stage shown in the DTG curve, which might be related to the oxidation of residues created in the second stage. The fiber/C1 surface featured an evident open hole (Figure 2d), thus showing that the degradation of the epoxy in the fiber/epoxy composite in the presence of oxygen was accompanied by the volatilization of carbon–containing components. The C/O ratios of the fiber/epoxy composite, fiber/C1, and fiber/C2 were determined to be 1.14, 0.81 and 1.99, respectively (Figure 3a). The volatilization of the epoxy resin under N_2_ conditions mostly involved the volatilization of small oxygen–containing molecules, thus resulting in the retention of the bulk of the carbon. The reduction in the C/O ratio of fiber/C1 suggested that the pyrolysis of epoxy resin in the oxidizing atmosphere would result in the in situ heterogeneous oxidation of epoxy resin, as well as small molecule volatilization. This result was consistent with the findings of the TG investigation.

SEM mapping for fiber/C1 and fiber/C2 was performed (Figure 3c and Figure S7), and the main elements of fiber/C1 and fiber/C2 were still C, N, O, Si, and Ca. Due to glass fiber’s high thermal stability, the Si, Ca, and O signals may have adapted better to the fibers’ structures, as the fibers’ structures were still intact. Furthermore, the N signals on the fibers’ surfaces, the debris, and substrate may be acquired for the N imaging of fiber/epoxy@C1 and fiber/epoxy@C2. The N signal on the surface of the glass fibers and debris was substantially greater than the N signal on the substrate, thus suggesting that the fibers/epoxy had a tiny quantity of N, and they were still well preserved after pyrolysis. N and C have extremely similar atomic numbers; thus, a modest quantity of N doping might greatly improve the electrical and mechanical properties of carbon materials.

FTIR was used to further analyze the chemical structure of pyrolysis products (Figure 3b). C–H stretching vibrations were relevant for the peaks at 2959, 2924, and 2866 cm^−1^ (–CH_3_ and –CH_2_). Peaks at 1606 and 1507 cm^−1^ were linked to aromatic ring C=C stretching vibrations, and the absorption peak in the 1300–1100 cm^−1^ range was related to ether’s C–O stretching vibration. Moreover, the peak at 830 cm^−1^ was attributed to aromatic C–H out–of–plane deformation vibrations and the precise peak variations of fiber/C1 and fiber/C2 in the 915 cm^−1^ epoxy ring were not discernible. Fiber/C1 showed a typical C=O peak at 1723 cm^−1^, and the spectral band of 1300~1100 cm^−1^ widened and intensified, thus showing that additional oxygen–containing compounds were formed during the degradation of epoxy resin in an O_2_ environment. The intensity of the C–H stretching vibration in the range of 2800–3000 cm^−1^ of fiber/C1 considerably decreased or almost vanished; however, the peak in the fiber/C2 range remained, thus showing that carbon compounds were lost under aerobic circumstances and retained in the absence of O_2_.

The typical peaks of fiber/C1 and fiber/C2 in the XRD spectra clearly disappeared at around 20°, but the characteristic diffraction peaks of amorphous SiO_2_ remained (Figure 1c). Furthermore, the peak strength of fiber/C2 increased at 23° as a result of the cross–linking/condensation reaction during the decomposition process, which generated carbon rich compounds with higher disorders.

**Figure 4:**
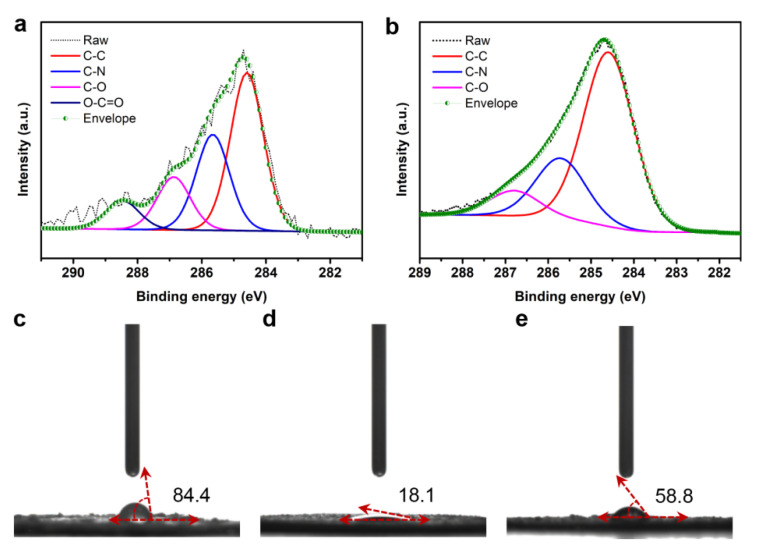
XPS survey spectra of C 1s of fiber/C1 (**a**), and fiber/C2 (**b**); water contact angle of the fiber/epoxy composite (**c**), fiber/C1 (**d**), and fiber/C2 (**e**).

In the XPS spectra of C 1s, both fiber/C1 and fiber/C2 had C–C/H, C–N and C–O, whereas the atomic concentration of C–O in fiber/C2 was 9.7%; this was substantially less than the 13.1% found in fiber/C1 (Figure 4a,b). Furthermore, there was a characteristic C=O peak in fiber/C1. These findings supported the typical thermal degradation variations between the fibers/epoxy in aerobic and anaerobic environments.

Previous characterizations indicated that the pyrolysis of fibers/epoxy under different atmospheres resulted in considerable changes in terms of the products’ oxygen content; this can also be confirmed by the water contact angle. The fibers/epoxy’s water contact angle was 84.4° (Figure 4c), thus showing that it had poor hydrophilic properties due to the hydrophobic resin covering the hydrophilic glass fibers. After pyrolysis, the water contact angles of fiber/C1 and fiber/C2 were lowered to 18.1 and 58.8°, respectively, thus demonstrating that pyrolysis would considerably increase hydrophilicity (Figure 4d,e). The increased hydrophilicity of fiber/C1 was caused by exposing the glass fibers and increasing the number of oxygenous sites. For fiber/C2, the pyrolysis of the epoxy resin resulted in the increased volatilization of oxygenated materials, and improved hydrophilicity was attributed to the exposure of the glass fibers.

### 3.4. Elastic Deformation

When inorganic materials are used as the reinforcing agent of a polymer, the interface between the inorganic materials and the polymer causes the inorganic materials to have a strong interfacial bondage in the polymer network; this causes both rigidity and toughness and can greatly improve tensile properties [40,41]. Different pyrolysis conditions can significantly alter the structure and surface groups of the epoxy of a fiber/epoxy composite, thus resulting in significant changes to the overall surface characteristics of the fibers/epoxy. Here, the glass fibers/resin were heat treated, using simple heat treatments to control its hydrophilic and hydrophobic properties, in order to achieve compatibility with the organic polymer and create flexible composite materials. As a result, the elastic deformation of fiber/epoxy@FC, fiber/C1@FC, and fiber/C2@FC differed when the fiber/epoxy composite was used as the reinforcing agent for SBR before and after pyrolysis.

**Figure 5:**
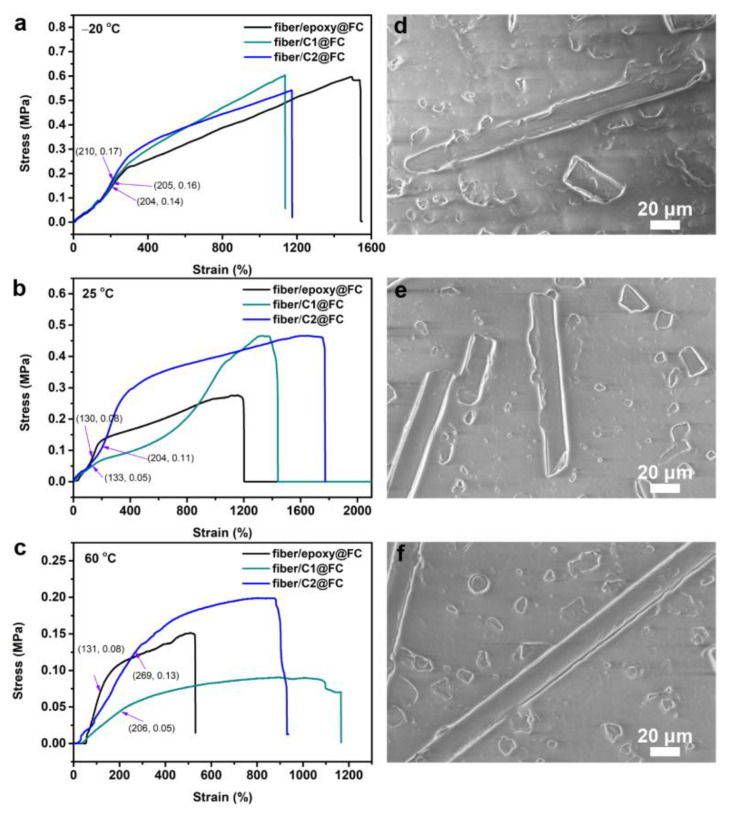
Stress–strain curves of fiber/epoxy@FC, fiber/C1@FC, and fiber/C2@FC at different temperatures: −20 °C (**a**), 25 °C (**b**), and 60 °C (**c**); SEM images of fiber/epoxy@FC (**d**), fiber/C1@FC (**e**), and fiber/C2@FC (**f**).

At ambient temperatures, the hardness of the fiber/epoxy@FC, fiber/C1@FC, and fiber/C2@FC were all less than 5 HA, with no significant difference. Their tensile properties, however, changed significantly at various temperatures. During the elastic stage, the strain of fiber/epoxy@FC, fiber/C1@FC, and fiber/C2@FC at −20 °C revealed a variation trend of fiber/C2@FC > fiber/C1@FC > fiber/epoxy@FC (Figure 5a); however, the differences were insignificant. At ambient temperatures, the highest strains of fiber/epoxy@FC, fiber/C1@FC, and fiber/C2@FC during the elastic stage were 130, 133, and 204%, respectively (Figure 5b), thus suggesting that fiber/C2 could improve the tensile properties of the flexible SBR composites significantly. Furthermore, when the temperature was raised to 60 °C, the greatest strains of fiber/epoxy@FC, fiber/C1@FC, and fiber/C2@FC were 131, 206, and 269%, respectively, during the elastic stage (Figure 5c).

As previously indicated, the glass fibers in the fiber/epoxy composite were covered with epoxy resin, and their surfaces conformed to the epoxy resin’s properties. Although fibers/epoxy have high hydrophobic properties, the larger epoxy resin granules and the disordered structure of the fiber/epoxy composite made it difficult to form a homogenous composite system with liquid paraffin and SBR (Figure 5d). In contrast, the epoxy resin formed more tiny granules following heat treatment; therefore, fiber/C1 and fiber/C2 had good dispersion performance in the formed composites (Figure 5e,f). The fiber/C2 surface was a hydrophobic carbon material structure and it had a stronger relationship with other components (liquid paraffin and SBR) than fiber/C1. The DSC curves of fiber/epoxy@FC, fiber/C1@FC, and fiber/C2@FC at −30~80 °C revealed that as the temperature rose, they assumed an overall exothermic state (Figure S8), thus implying that the temperature increase caused a stress release in these composites. The heat flow per unit mass sequence was fiber/C2@FC < fiber/C1@FC < fiber/epoxy@FC, thus revealing that fiber/C2@FC had less stress concentration. This also demonstrated that fiber/C2@FC was more compatible with SBR and liquid paraffin. In addition, these three composites showed minor endothermic peaks at 88 °C, thus indicating that a vitrification transition happened at around 80 °C. In conclusion, fiber/C2 was more evenly dispersed in fiber/C2@FC and had better compatibility with SBR and liquid paraffin than the fiber/epoxy composite; therefore, the best tensile properties for fiber/C2@FC occurred below 80 °C.

### 3.5. A Feasible Route towards Industrial Application

**Figure 6:**
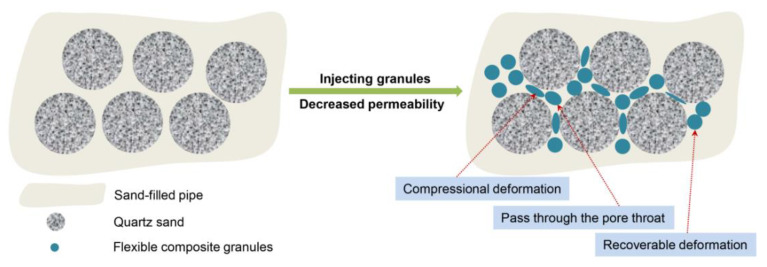
Schematic of flexible composite granules for plugging pore throats.

At high temperatures of up to 60 °C, the flexible composite in this investigation still showed a strain performance of up to 200%, thus indicating that it has potential utility as a plugging agent for large formation channels; therefore, the sand–filled model was adopted to investigate the plugging ability of fiber/C2@FC granules for large channels. The fiber/C2@FC granules were injected into the sand–filled pipe and aged for 3 days in a 120 °C oven. As shown in Table 2, the sealing rate was 67.1% with an injection volume of 3.0 pore volume (PV), thus proving that the fiber/C2@FC granules had a high plugging ability for large channels. When the injection volume was raised to 4.0 PV, the plugging rate increased to 81.3%. When the injection volume was increased to 5.0 PV, the plugging rate did not noticeably change. The effect of mineralized water on the plugging performance of the fiber/C2@FC granules was explored to further imitate the formation environment. The plugging rate and breakthrough pressure of the fiber/C2@FC granules did not fluctuate considerably when the injection volume was 5.0 PV and the salinity increased from 50 to 200 mg L^−1^, thus suggesting that the fiber/C2@FC granules demonstrated good salt resistance. Furthermore, after 3 months in the mineralized water of the swelling experiment, the flexible composite granules still did not display any swelling phenomena, thus showing that the flexible composite granules exhibited better aging resistance. The flexible granules were thermally stable at 120 °C and resilient at 60 °C, thus allowing them to fill holes via retention and deformation processes (Figure 6) [42,43]. A higher plugging rate was especially useful for improving the displacement agent’s entrance into the oil–bearing channel that had a low water content, which had a substantial application value in terms of increased oil recovery.

## 4. Conclusions

Developing novel methods of fiber/epoxy recycling and usage is crucial for promoting global low–carbon growth and environmental protection. The pyrolysis mechanism of fibers/epoxy from scrap WTB was explored in this study, as well as the characteristics of fibers/epoxy as reinforcing components in flexible composites. The following results were obtained.

(1) The glass fibers in the fiber/epoxy composite remained relatively stable throughout the pyrolysis process, and the pyrolysis of epoxy resin was connected to the pyrolysis atmosphere. The early degradation of epoxy resin in air was substantially faster than in nitrogen, and the pyrolysis of epoxy resin in air cannot be described simply through pyrolysis or combustion models.

(2) The water contact angles of the fibers/epoxy after heat–treatment in the oxidizing and inert atmospheres were 18.1 and 58.8°, respectively. The pyrolysis of epoxy resin in an oxidizing atmosphere could result in more oxygen–containing sites, but pyrolysis in an inert atmosphere would result in the removal of most of the O and the formation of an amorphous carbon structure.

(3) SBR and liquid paraffin were more compatible with fiber/C2 than fiber/epoxy and fiber/C1; therefore, fiber/C2@FC demonstrated the greatest tensile performance in the temperature range of −20 to 60 °C, with the tensile performance in the first elastic stage reaching 200% at 60 °C.

(4) Fiber/C2 composite granules could achieve an 81.1% plugging rate at 5.0 PV, and the plugging performance was essentially unaffected by water salinity, thus indicating that they have a high application potential in plugging large channels during oil and gas extraction.

(5) Pyrolysis offers a novel approach for the value–added exploitation of fibers/epoxy from scrap WTB, as well as a new method for large–scale fiber/epoxy applications in flexible composite materials.

## Figures and Tables

**Table 1:** Thermal decomposition data of the fibers/epoxy under different atmospheres.

Atmosphere	Temperature (°C)					(dm/dt)_max_ (% min^−1^)	
	T_0.05_	T_0.11_	T_0.22_	T_max1_	T_max2_	T_max1_	T_max2_
Air	292.9	356.4	634.0	292.5	538.4	1.81	0.74
N_2_	328.4	354.8	–	345.6	658.8	2.48	0.15

**Table 2:** Plugging rate of fiber/C2@FC granules with different injection volumes.

PV	NaCl (g L^−1^)	Permeability (mD)		Plugging Rate (%)
		Before the Injection of Fiber/C2@FC Granules	After the Injection of Fiber/C2@FC Granules	
3.0	0	1987	653	67.1
4.0	0	1931	361	81.3
5.0	0	2022	354	82.5
5.0	50	2236	422	81.1
5.0	100	2214	440	80.1

## Data Availability

The data presented in this study are available on request from the corresponding author.

## Supplementary Materials

The following supporting information can be downloaded at: https://www.mdpi.com/article/10.3390/polym14245408/s1, Figure S1: Scrap WTB: bulk (a), ground powder (fiber/epoxy) (<20 mesh), thermal treatments with air (c) and N2 (d). Figure S2: The length distribution of fibers in fiber/epoxy composites powder from scrap WTB (<20 mesh). Figure S3: Wide scan of XPS survey spectra of fiber/epoxy. Figure S4: XPS survey spectra of O 1s of fiber/epoxy. Figure S5: XPS survey spectra of N 1s of fiber/epoxy. Figure S6: XPS survey spectra of Ca 2p of fiber/epoxy. Figure S7: SEM imaging of fiber/C1. Figure S8: DSC curves of fiber/epoxy@FC, fiber/C1@FC, and fiber/C2@FC.
