# Supplementary material for: Zero–Waste Recycling of Fiber/Epoxy from Scrap Wind Turbine Blades for Effective Resource Utilization
Source: Polymers (Basel). 2022 Dec 10;14(24):5408. doi: 10.3390/polym14245408 (PMC9785109; doi:10.3390/polym14245408)
Supplement: Supplementary file 1 [file polymers-14-05408-s001.zip › polymers-2088074-Supplementary.pdf]

Zero-Waste Recycling of Fiber/Epoxy from Scrap Wind Turbine Blades for Effective Resource Utilization

Chunbao Du[1,2,*], Ge Jin[1], Lihui Zhang[3], Bo Tong[3], Bingjia Wang[3], Gang Zhang[4,*], Yuan Cheng[5,6,*]

[1] College of Chemistry and Chemical Engineering, Xi'an Shiyou University, Xi'an 710065, P. R. China

[2] Shaanxi Engineering and Technology Research Center of Green Low–Carbon Energy Materials and Processes, Xi'an 710065, China

[3] Xi'an Thermal Power Research Institute Co., Ltd., Xi'an 710054, P. R. China

[4] Institute of High Performance Computing, A*STAR, Singapore 138632, Singapore

[5] Monash Suzhou Research Institute, Monash University, Suzhou Industrial Park, Suzhou 215000, P. R. China

[6] Department of Materials Science and Engineering, Monash University, Clayton, VIC 3800, Australia

[*] Corresponding author. E-mail: duchunbao@xsyu.edu.cn, zhangg@ihpc.a-star.edu.sg, yuan.cheng@monash.edu

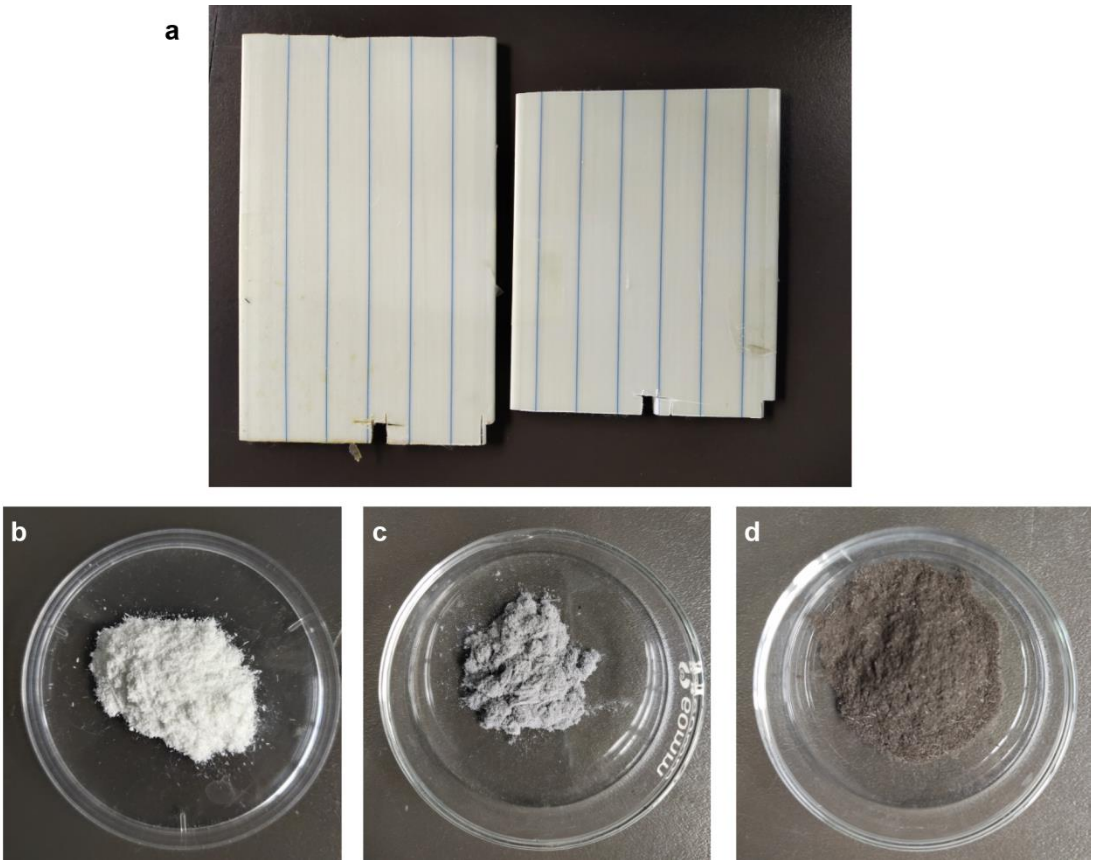

Figure S1. Scrap WTB: bulk (a), ground powder (fiber/epoxy) (<20 mesh), thermal treatments with air (c) and N_2 (d).

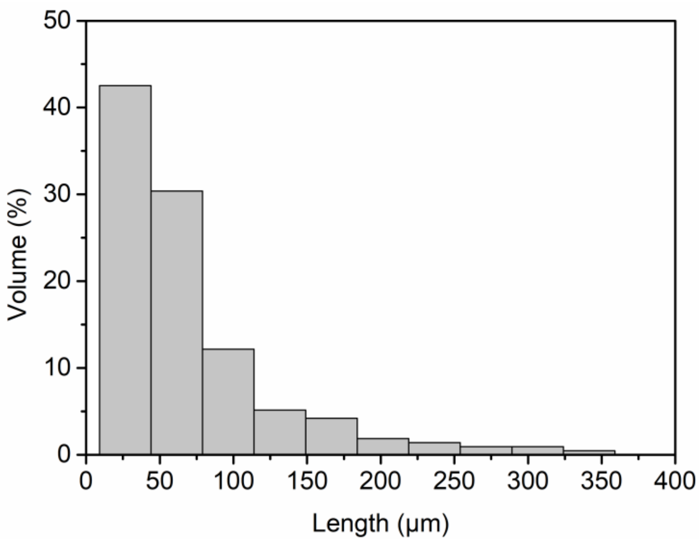

Figure S2. The length distribution of fibers in fiber/epoxy composites powder from scrap WTB (< 20 mesh).

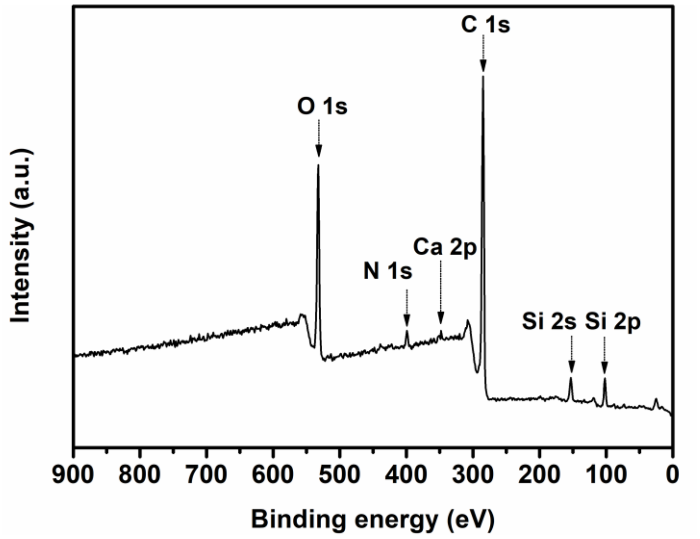

Figure S3. Wide scan of XPS survey spectra of fiber/epoxy.

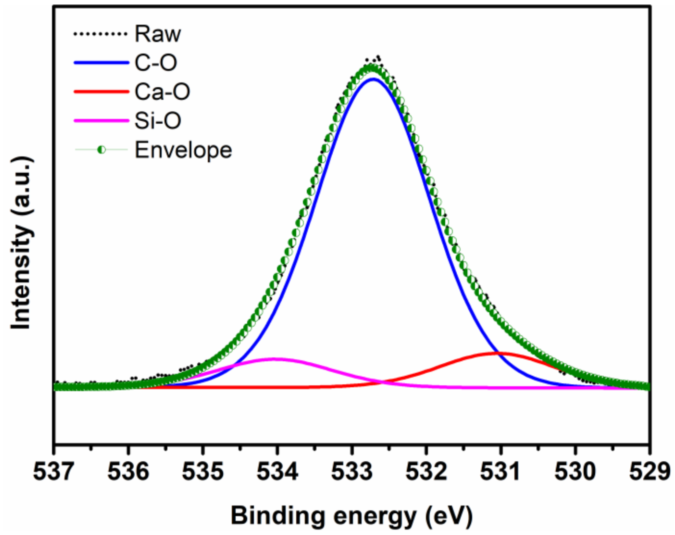

Figure S4. XPS survey spectra of O 1s of fiber/epoxy.

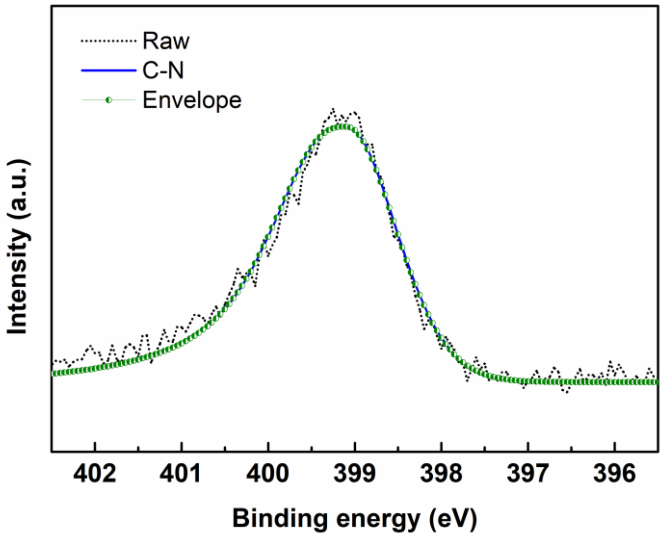

Figure S5. XPS survey spectra of N 1s of fiber/epoxy.

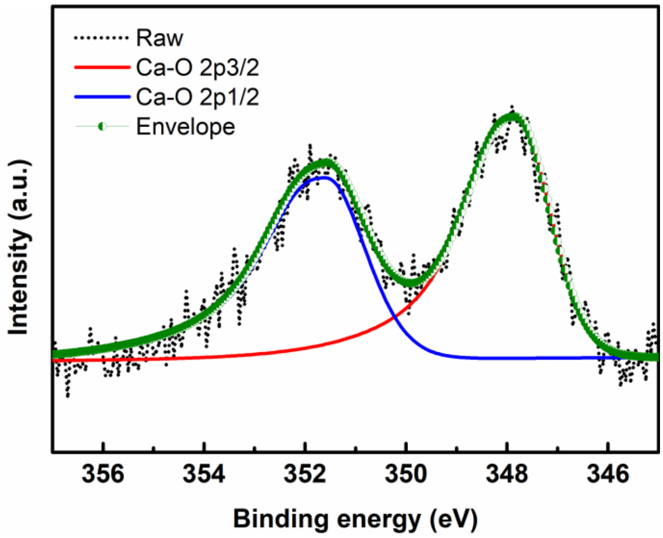

Figure S6. XPS survey spectra of Ca 2p of fiber/epoxy.

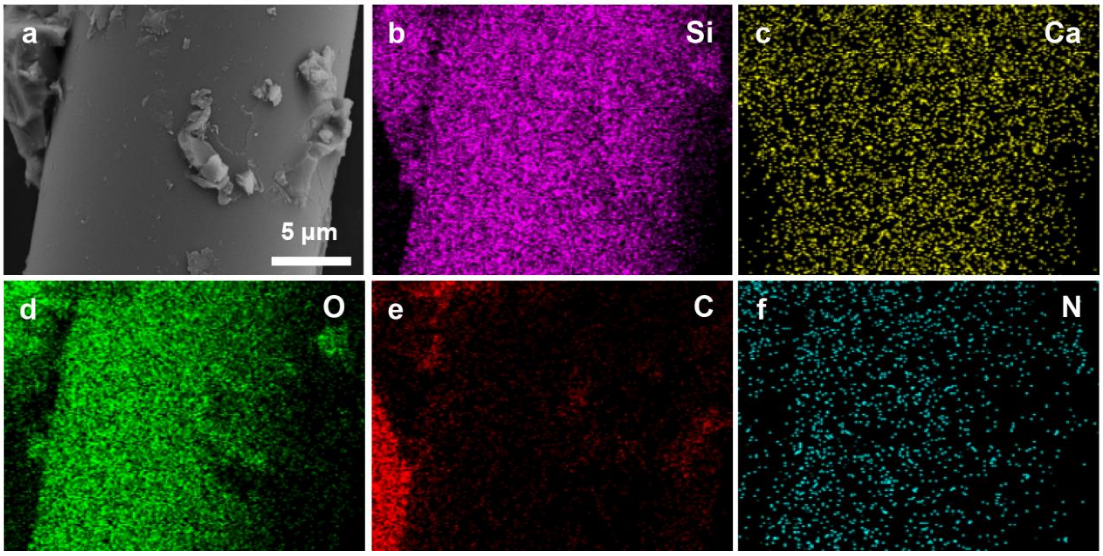

Figure S7. SEM imaging of fiber/C1.

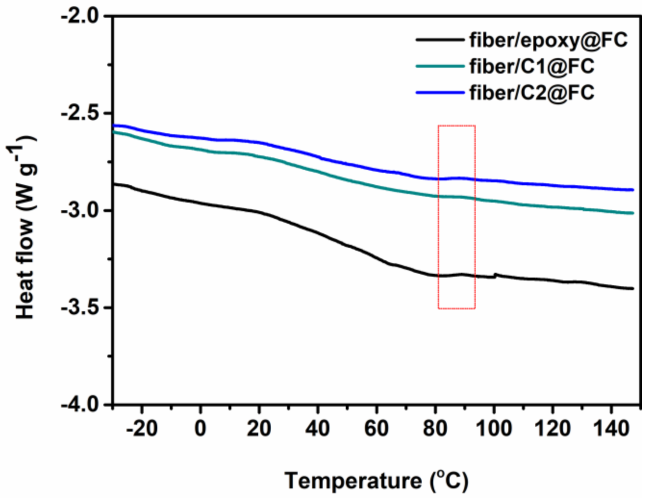

Figure S8. DSC curves of fiber/epoxy@FC, fiber/C1@FC, and fiber/C2@FC.
